# Microbiota and COVID-19: Long-term and complex influencing factors

**DOI:** 10.3389/fmicb.2022.963488

**Published:** 2022-08-12

**Authors:** Jiaqi Gang, Haiyu Wang, Xiangsheng Xue, Shu Zhang

**Affiliations:** ^1^Department of Emergency, The First Affiliated Hospital of Zhengzhou University, Zhengzhou, China; ^2^Department of Oncology, Xiuwu County People’s Hospital, Jiaozuo, China; ^3^Department of Infectious Diseases, The First Affiliated Hospital of Zhengzhou University, Zhengzhou, China

**Keywords:** COVID-19, SARS-CoV-2, microbiota, ACE2, diagnostic model, microbiota transplantation, traditional Chinese medicine

## Abstract

The coronavirus disease 2019 (COVID-19) is an infectious disease caused by severe acute respiratory syndrome coronavirus 2 (SARS-CoV-2). According to the World Health Organization statistics, more than 500 million individuals have been infected and more than 6 million deaths have resulted worldwide. Although COVID-19 mainly affects the respiratory system, considerable evidence shows that the digestive, cardiovascular, nervous, and reproductive systems can all be involved. Angiotensin-converting enzyme 2 (AEC2), the target of SARS-CoV-2 invasion of the host is mainly distributed in the respiratory and gastrointestinal tract. Studies found that microbiota contributes to the onset and progression of many diseases, including COVID-19. Here, we firstly conclude the characterization of respiratory, gut, and oral microbial dysbiosis, including bacteria, fungi, and viruses. Then we explore the potential mechanisms of microbial involvement in COVID-19. Microbial dysbiosis could influence COVID-19 by complex interactions with SARS-CoV-2 and host immunity. Moreover, microbiota may have an impact on COVID-19 through their metabolites or modulation of ACE2 expression. Subsequently, we generalize the potential of microbiota as diagnostic markers for COVID-19 patients and its possible association with post-acute COVID-19 syndrome (PACS) and relapse after recovery. Finally, we proposed directed microbiota-targeted treatments from the perspective of gut microecology such as probiotics and prebiotics, fecal transplantation and antibiotics, and other interventions such as traditional Chinese medicine, COVID-19 vaccines, and ACE2-based treatments.

## Introduction

Severe acute respiratory syndrome coronavirus 2 (SARS-CoV-2), the pathogen of coronavirus disease 2019 (COVID-19), is currently the seventh known coronavirus that can infect humans. It is highly contagious and the population is generally susceptible. This characteristic has caused SARS-CoV-2 to spread across the world and has made a huge difference in global politics and the economy. Currently, the global epidemic has not been completely controlled, and has recurred repeatedly. SARS-CoV-2 has evolved various variants such as alpha, beta, gamma, delta, and omicron. COVID-19 is most often manifested as fever, irritating dry cough, fatigue, loss of smell or taste, and some will have diarrhea, headache, etc. (Chen et al., 2020; World Health Organization, 2022). In addition, the disease will also have long-term effects on people, leaving multiple systemic sequelae, such as heart and kidney damage, thromboembolism, persistent neurocognitive deficits, anxiety, depression, and muscle weakness. COVID-19 mainly affects the respiratory system, but studies have confirmed that the SARS-CoV-2 can still be detected in fecal samples after the respiratory symptoms disappear and once nasopharyngeal tests are negative, indirectly indicating that the gastrointestinal tract may also be another site of infection (Wu et al., 2020; Xiao et al., 2020; Xu Y. et al., 2020; Zhou J. et al., 2020). In parallel, some patients experience gastrointestinal symptoms such as diarrhea, abdominal pain, vomiting, and nausea further confirming this view (Pan L. et al., 2020). Related reports have proposed direct evidence of active replication of SARS-CoV-2 in the intestine of the human (Qian et al., 2021) and non-human primate models (Jiao et al., 2021). A recent study further has also shed light on the underlying mechanisms by which SARS-CoV-2 infection causes gastrointestinal symptoms. The SARS-CoV-2 spike protein activates the Ras-Raf-MEK-ERK-VEGF pathway in intestinal epithelial cells and promotes the production of vascular endothelial growth factor (VEGF), which leads to vascular permeability and inflammation (Zeng F.M. et al., 2022).

Current diagnostic approaches are based on three aspects: epidemiological history, clinical manifestations, and related auxiliary examinations (Demertzis et al., 2020; Mao et al., 2020; Troyer et al., 2020). Among these, reverse transcription-polymerase chain reaction (RT-PCR) molecular tests are widely used in clinics due to the high accuracy and the convenience of sampling, and are currently the gold standard for diagnosing COVID-19. However, the incubation period infections, differences in viral load in different samples, unqualified reagents, and non-standardized testing methods can all lead to false negatives, and failure to detect confirmed cases in timely manner, can contribute to widespread transmission. Other detection methods, such as viral antigen detection and human antibody detection, have advantages and limitations. Antigen detection is cheap, fast, simple to operate, and can be performed outside the laboratory, while the sensitivity is low. Antibody tests are typically used for retrospective analysis and provide indirect evidence of SARS-CoV-2 infection two weeks after symptoms appear. However, it is unable to distinguish between natural infection and vaccine-induced immunity (Peeling et al., 2022). Furthermore, there is no specific treatment for COVID-19. The conventional treatment plan includes antiviral treatment, respiratory support, nutritional support, immunotherapy, and other symptomatic treatment. Therefore, more effective diagnoses and treatments of COVID-19 must be urgently identified.

The microbiota, which consists of bacteria, fungi, viruses, and archaea, defines the totality of microorganisms that exist inside and on the human body. The microbiota is currently a hot topic among researchers. Growing studies have shown that the microbiota plays a key role in human health. Stable microbiota can promote the absorption and digestion of nutrients, improve immune function of the body, and synthesize enzymes, vitamins, and other essential substances. Additionally, the microbiota can have an effect on disease progression, improve treatment effects, and also serve as diagnostic tools, such as for liver cirrhosis (Qin et al., 2014), type 2 diabetes (Qin et al., 2012), and autism (Kang et al., 2017). For example, *Helicobacter pylori* infection has been confirmed to be related to gastritis, and long-term infection is a high-risk factor leading to gastric cancer (Moss, 2017). Specific gut microbiome signatures could serve as a non-invasive diagnostic model for liver cirrhosis (Oh et al., 2020).

Therefore, many scholars have turned their attention to the relationship between the microbiota and COVID-19 during the pandemic. The Microbiome Centers Consortium COVID Committee proposes an initiative to facilitate microbiome research in the context of the COVID-19 pandemic in a coordinated, assisted manner (Microbiome Centers Consortium Covid Committee, 2020). Numerous studies have shown that SARS-CoV-2 infection has adverse effects on the respiratory, intestinal, and oral microbiota of the body, manifested primarily in the decline of microbial diversity and uniformity, the reduction of beneficial symbiotic bacteria, and the increase in opportunistic pathogens. This microbiota dysbiosis may persist after recovery from COVID-19 (Zuo et al., 2020b; Gao et al., 2021; Ren et al., 2021). Imbalance and invasion of the microbiota may disrupt the function of the body barrier and may weaken host immunity. This review systematically describes the microbiota changes in the different parts of patients with COVID-19 and the possible role and mechanisms involving the microbiota in the severity of the disease, its diagnosis and treatment, and in predicting prognosis to further strengthen the understanding of COVID-19 and to provide novel insight for prevention strategies.

## Microbiota dysbiosis in COVID-19

### Composition and changes in the gut microbiota

#### Composition and changes in the gut bacterial microbiota

Zuo et al. (2020b) conducted a shotgun metagenomic analysis using fecal samples from 15 patients with COVID-19 from their initial hospitalization to discharge and concluded that the altered intestinal environment manifested an enrichment of pathogenic opportunistic bacteria and the reduction of beneficial symbiotic bacteria. The Firmicutes phylum was primarily related to the severity of COVID-19, among which *Coprobacillus, Clostridium ramosum*, and *Clostridium hathewayi* were positively correlated with the severity of COVID-19, and, in contrast, *Faecalibacterium prausnitzii, Faecalibacteriu, and Alistipes onderdonkii of Bacteroides phylum* were negatively correlated with disease severity (Zuo et al., 2020b). Another study exploring the microbiota and the severity of COVID-19 found that compared to patients with mild COVID-19, moderate to severe patients had a lower *Firmicutes/Bacteroidetes* ratio, a higher abundance of *Proteobacteria*, and a lower abundance of *Lachnospiraceae* and *Actinobacteria* (Moreira-Rosário et al., 2021). Subsequently, a study revealed that *Collinsella aerofaciens, Collinsella tanakaei, Streptococcus infantis* were more abundant in fecal samples of highly infectious SARS-CoV-2, while the short-chain fatty acid producing bacteria *Parabacteroides merdae, Alistipes onderdonkii, Bacteroides stercoris*, *and Lachnospiraceae bacterium 1_1_57FAA* were more abundant in fecal samples with low-to-none SARS-CoV-2 infection (Zuo et al., 2021a). A recent study found that *Ruminococcus gnavus, Eggerthella, Coprobacillus, Lachnospiraceae bacterium 2_1_58 FAA, Clostridium ramosum* were enriched in COVID-19 patients, on the contrary *Alistipes_sp_AP11, Roseburia intestinalis, Eubacterium hallii, Alistipes indistinctus, Coprobacter fastidiosus, Alistipes shahii* were reduced, it also proved in mice model that infection with SARS-CoV-2 reduced the microbial diversity (Cao et al., 2021). This conclusion is consistent with the conclusions of Zuo et al. (2020b) on the severity of microbes and COVID-19. Using *16S rRNA* gene sequencing, Reinold et al. (2021) determined that the relative abundance of *Bifidobacterium*, *Collinsella*, and *Streptococcus* was low in SARS-CoV-2-positive patients, while the abundance of *Bacteroidetes* and *Enterobacteriaceae* was higher. Furthermore, the depletion of *Faecalibacterium* and *Roseburia* has also been described in severe/critical patients. When comparing these studies, the common feature is that COVID-19 patients present significantly reduced microbial diversity, decreased abundance of beneficial commensal bacteria, and increased abundance of opportunistic pathogens compared to healthy individuals. This microbiota dysbiosis may persist even after the respiratory clearance of SARS-CoV-2. Some studies have found that at the phylum level, *Proteobacteria* was enriched, and the abundance of *Actinobacteria* decreased in COVID-19 individuals. The abundance of *Bifidobacterium, Colinella*, and *Streptococcus* at the genus level was lower in COVID-19 individuals (Moreira-Rosário et al., 2021; Reinold et al., 2021; Yeoh et al., 2021). However, in contrast to these findings, other studies have found that the relative abundance of *Actinobacteria* was higher (Gu et al., 2020), and the abundance of members of the genera *Bifidobacterium, Streptococcus*, and *Lactobacillus* increased (Tao et al., 2020). The possible interference of ethnicity, dietary habits, COVID-19 severity, and treatment measures cannot be ruled out.

Additionally, researchers found that the intestinal microbiome is related to COVID-19 complications and mortality. A prospective cohort study collected the fecal and saliva samples from COVID-19 patients, post-COVID-19 patients, pneumonia controls, and asymptomatic controls. After targeted *16S rRNA* gene sequencing, *F. prausnitzii* and alpha-diversity were inversely associated with the number of complications, specific complications were related to certain bacteria, while the mortality of patients with COVID-19 can be predicted based on the characteristics of intestinal bacteria (Schult et al., 2022). In parallel, Cao et al. (2021) analyzed differences in gene expression in intestinal epithelial cells following SARS-CoV-2 infection through transcriptomic analysis. The authors concluded that compared to infected (vaccinated) mice, immune-related genes such as ZEB1 and pleiotropic APOE, which were related to SARS-CoV-2 infection or severity, were enriched in infected (non-vaccinated) mice. These findings raised many questions that need further investigation, including the changes in microbial gene function during COVID-19 and the impact of antibiotic treatment on the composition of the bacterium/virome.

Judging from the data collected to date, despite the several studies examining changes in the gut microbiota of patients with COVID-19 and their relationship with the severity of the disease, these studies preset limitations such as (i) a definition of the characteristics of the gut microbiota before SARS-CoV-2 infection and after SARS-CoV-2 clearance, (ii) the disease stage of each patient is unclear at the time of sample collection, and (iii) the small sample size. To resolve these problems, some researchers turned to animal experiments and conducted research on non-human primates (macaques) (Sokol et al., 2021) and hamsters’ models (Sencio et al., 2022). Both models found a decrease in the abundance of several members of then fatty acid-producing *Firmicutes* phylum, including *Ruminococcaceae* and *Lachnospiraceae*, in line with what was observed in humans. When comparing changes in the gut microbiota before and after SARS-CoV-2 infection, it was also confirmed in animal models that changes in the composition of the gut microbiota are related to COVID-19. Despite the shortcomings of these studies, the intricate interaction between COVID-19 and the gut microbiota is undeniable.

#### Composition and changes in the gut mycobiota

Fungi account for less than 1% of the human microbiome but play an essential role in controlling the inflammatory response and in regulating the homeostasis of the microbial community (Kumamoto, 2016; Li et al., 2019; Rawson et al., 2021). Hoffmann et al. (2013) detected 66 fungal genera in fecal samples of healthy individuals, the highest detection rate being for *Saccharomyces*, followed by *Candida* and *Cladosporium*. In stool samples taken at all time points in patients with COVID-19, *Candida albicans, Candida auris, and Aspergillus flavus* were higher than in controls. Furthermore, COVID-19 patients presented greater mycobiome heterogeneity during hospitalization, and mycobiome dysregulation persisted even after SARS-CoV-2 was cleared in nasopharyngeal samples and respiratory symptoms disappeared, as revealed by a study of the fecal fungal microbiome in patients with COVID-19 (Zuo et al., 2020a). *Candida albicans* is the main inducer of antifungal Th17 cells. Furthermore, total *Candida albicans* and Th17 cells increase in the state of intestinal inflammation (Bacher et al., 2019). *Candida* is abundant in inflammatory bowel disease, and the pathogenesis of celiac disease is associated with *Candida* (Sokol et al., 2017; Wu X. et al., 2021). Based on these studies, it is reasonable to speculate that the abundance of *Candida albicans* during hospitalizations in patients with COVID-19 may be associated with intestinal inflammation. Similarly, another study of the intestinal mycobiota in patients with COVID-19 and H1N1 found that, compared to healthy controls, patients with COVID-19 had an increased intestinal fungal load, the abundance of fungal species with crucial functions was relatively reduced and the relative abundance of pathogenic opportunistic fungi increased. Moreover, *Ascomycota and Basidiomycota* members were nearly exhausted (Lv et al., 2021). A cross-sectional study evaluated the characteristics of the intestinal mycobiota of COVID-19 patients with different severities. Unlike the findings in the bacterial microbiota, which typically appears as a mixture of various species, a single species dominated the fungal gut microbiota in most critically ill COVID-19 patients. Compared to patients with mild COVID-19 illness, the relative abundance of *Ascomycota* increased, while the diversity, evenness, and richness of gut mycobiota decreased in patients with severe/critical illness (Reinold et al., 2022). The *Ascomycota phylum and Basidiomycota* are major components of the human gut mycobiota (Wu et al., 2019; Fiers et al., 2020), which really indicates that the normal intestinal fungal community is destroyed. The reason for several conflicting conclusions of these studies is not apparent, but may be associated with age, sex, and eating habits (Hoffmann et al., 2013; Strati et al., 2016; Fiers et al., 2020). Additional studies are needed to fully understand the intestinal mycobiota in COVID-19. Some scholars have proposed the influence of intestinal mycobiota on extraintestinal organs, and the existence of the gut-lung axis, gut-brain axis, etc., which may predict the severity and prognosis of the disease through intestinal fungi and their extraintestinal targets in the future (Wu X. et al., 2021). A cross-sectional study evaluated the characteristics of the intestinal mycobiota of COVID-19 patients with different severities. Unlike the findings in the bacterial microbiota, which typically appears as a mixture of various species, a single specie dominated the fungal gut microbiota in most critically ill patients.

#### Composition and changes in the gut virome

In addition to the bacterial microbiota and mycobiota, the virome is also a non-negligible component of the gut microbiome. The human gut virome is highly individualized and reveals high temporal stability (Shkoporov et al., 2019). It consisting of phages and eukaryotic viruses, of which phages represent more than 90%. Bacteriophages, eukaryotic viruses, and plant-derived viruses interact with symbiotic bacteria to maintain the intestinal barrier and regulate intestinal motility (Lopetuso et al., 2016; Guerin et al., 2018; Li Y. et al., 2021). A small body of literature has investigated the gut virome of patients with COVID-19. First, Cao and his colleagues probed the composition and variation of the enterovirus group in patients with COVID-19 and validated it in mouse experiments. It was concluded that bacteriophages (*Inoviridae* and *Microviridae*), plant-RNA virus cucumber green mottle mosaic viruses, and unclassified viruses were enriched in patients with COVID-19, and that antibiotic treatment did not affect the composition of enteroviruses (Cao et al., 2021). Subsequently, a study analyzed the virome of fecal DNA and RNA using metagenomic sequencing. The results showed that the SARS-CoV-2 infected fecal virus group had more genetic codes associated with inflammation, stress, and toxicity. SARS-CoV-2 infection had the most significant effect on enterovirus, while the effects of infection, sex, and age were not significant (Zuo et al., 2021b), as exemplified in the work by Cao et al. (2021). Eight DNA viruses were inversely correlated with the severity of COVID-19 disease and blood inflammatory markers, including CRP, LDH, and neutrophils (Zuo et al., 2021b), which showed that enteroviruses can mediate host immune responses to combat SARS-CoV-2 infection. Lu et al. (2021) concluded that the abundance of crass-like bacteriophages in COVID-19 patients decreased significantly compared to healthy controls. There were significant correlations between enteroviruses and bacterial communities, such as *Tectiviridae* and *Microviridae*, *Tectiviridae*, and *Bacteroidaceae*, the relative abundance ranges, although little varied, and the overall trend was similar (Lu et al., 2021). This conclusion substantiates what was found by Cao: in which three bacteria (*Bacteroides vulgatus, Faecalibacterium prausnitzii*, and *Ruminococcus gnavus*) and three microviridae bacteriophages played a key role in virus-bacterial community interactions (Cao et al., 2021). Although the virome has individual specificity, its structure and diversity correspond to the intestinal flora (Moreno-Gallego et al., 2019). First, bacteria can prevent phage adsorption through the biofilm, degrade phage nucleic acid and other anti-bacteriophages (Simmons et al., 2018; Rostøl and Marraffini, 2019). However, the activity of bacteriophages affects bacterial behavior, mediating gene transfer between bacteria in the host inflammatory state. Furthermore, there is a co-evolution of bacteriophages and bacteria in the gastrointestinal tract (De Sordi et al., 2019). OM-85, a bacterial lysate, can down-regulate SARS-CoV-2 receptors and inhibit intestinal epithelial SAR-COV-2 infection in patients with COVID-19 (Pivniouk et al., 2021). The gastrointestinal symptoms of COVID-19 are not caused by the invasion of intestinal receptors by SARS-CoV-2 alone but are the result of the complex interaction of the gut microbiota, virome, and immune barriers. The characteristics of the gut microbiota in COVID-19 patients are shown in Table 1.

**TABLE 1:** The characteristics of gut microbiota in COVID-19 patients.

Sample size	Microbiota species	Geographic location	Gut microbiota characteristics in COVID-19	Ref.
15 COVID-19 patients, 6 community acquired pneumonia patients, 15 healthy controls	Bacterial	Hong Kong, China	Enrichment of opportunistic pathogenic bacteria and reduction of beneficial symbiotic bacteria, the baseline abundance of *Clostridium ramosum, Coprobacillus*, and *Clostridium hathewayi* was correlated with COVID-19 severity, whilst *Alistipes onderdonkii* and *Faecalibacterium prausnitzii* abundance inverse correlated with the disease severity	Zuo et al., 2020b
100 COVID-19 patients, 79 non-COVID-19 controls	Bacterial	Hong Kong, China	Gut microbiota known to have immunomodulatory potential such as *Faecalibacterium prausnitzii, Eubacterium rectum*, and *Bifidobacterium* were depleted. After adjusting for antibiotic use and patients’ age, *F. prausnitzii* and Bifidobacterium bifidum were negatively correlated with the severity	Yeoh et al., 2021
9 COVID-19 children aged from 7 to 139 months, 14 age-matched healthy controls	Bacterial	Not provided	At phylum level *Bacteroidetes* and *Firmicutes* were significantly more abundant in the gut of children with COVID-19 than healthy controls. Pathogenic bacterium *Pseudomonas* dominated among the gut microbiota, and persistent exist during the COVID-19.	Xu R. et al., 2021
36 COVID-19 patients, 23 suspected patients, 72 healthy controls	Bacterial	Henan, China	At genus level *Akkermansla, Streptococcus, Enterococcus*, and *Bifidobacterium* were enriched in COVID-19 patients whereas *Pseudobutyrivibrio, Blautia, Faecalibacterium*, and *Bacteroides* were more abundant in healthy people	Ren et al., 2021
86 COVID-19 patients, 21 post COVID-19 patients, 11 pneumonia controls, 26asymptomatic controls	Bacterial	Germany	*Parabacteroides* was positively correlated with the severity of COVID-19, *Fusicatenibacter* was negatively correlated with disease severity. *F. prausnitzii* was inversely associated with the number of complications and mortality, the relative abundance of the genus *Alistipes* was increased with the number of complications	Schult et al., 2022
13 COVID-19 patients, 5 healthy controls	Bacterial	Beijing, China	*Ruminococcus gnavus, Coprobacillus, Eggerthella, Lachnospiraceae bacterium 2_1_58 FAA, Clostridium ramosum*, and *Eggerthella lenta* were enriched in COVID-19 patients, while *Alistipes_sp_AP11, Alistipes indistinctus, Eubacterium hallii, Roseburia intestinalis, Burkholderiales bacterium 1_1_47, Coprobacter fastidiosus, Eubacterium eligens, Bacteroides salyersiae, Odoribacter splanchnicus, Alistipes shahii, Ruminococcus bromii, and Bacteroides massiliensis* were significantly depleted	Cao et al., 2021
115 COVID-19 patients, (mild, 19; moderate, 37; or severe 59)	Bacterial	Portuguese	The abundance of *Actinobacteria* and *Lachnospiraceae* was lower, and the abundance of *Proteobacteria* was higher in moderate and severe COVID-19 patients than in mild COVID-19 patients.	Moreira-Rosário et al., 2021
117 patients infected with SARS-CoV-2, 95 SARS-CoV-2 negative patients	Bacterial	German (98% Caucasian ethnicity)	In SARS-CoV-2 positive patients, the abundance of *Bacteroidetes* and *Enterobacteriaceae* was higher, and the abundance of several genera such as *Bifidobacterium, Streptococcus*, and *Collinsella* was lower.	Reinold et al., 2021
62 COVID-19 patients, 33 seasonal flu patients, 40 healthy controls	Bacterial	Hefei, China	Compared with healthy controls, members of the gen*era Streptococcus, Lactobacillus, Clostridium, and Bifidobacterium* was increased in COVID-19, while members of the general *Bacteroidetes, Faecalibacterium, Roseburia, Parabacteroides*, and *Coprococcus* was decreased.	Tao et al., 2020
30 COVID-19 patients, 9 community acquired pneumonia patients, 30 healthy controls	Fungal	Hong Kong, China	Enrichment of opportunistic fungal pathogens, *Candida aureus*, *Candida albicans*, and *Aspergillus flavus* during the disease course	Zuo et al., 2020a
67 COVID-19 patients, 35 H1N1 infected patients, 48 healthy controls	Fungal	Zhejiang, China	Increased fungal load and enrichment of some opportunistic pathogenic fungi. Ascomycota (such as *Penicillium polonicum, Penicillium citrinum, and Aspergillus* with its five species) and Basidiomycota (such as *Malassezia yamatoensis, Rhodotorula mucilaginosa, Moesziomyces aphidis*) were depleted	Lv et al., 2021
30 COVID-19 patients (21 non-severe COVID-19, 9 developing severe/critical COVID-19), 23 healthy controls	Fungal	German (mainly Caucasian ethnicity)	Increased abundance of *Ascomycota* phylum and the genus *Bipolaris*, and reduced fungal gut microbiota diversity, evenness and richness in severe/critical COVID-19 compared with non-severe COVID-19.	Reinold et al., 2022
13 COVID-19 patients, 5 healthy controls	Virome	Beijing, China	Enrichment of bacteriophages (*Inoviridae* and *Microviridae*), plant-RNA virus cucumber green mottle mosaic viruses, and unclassified viruses. 14 Microviridae phages, one Podoviridae phage, one Inoviridae phage, and one unclassified virus were enriched in severe COVID-19 cases	Cao et al., 2021
98 COVID-19 patients, 78 non-COVID-19 controls matched for gender and co-morbidities	Virome	Hong Kong, China	Enrichment of environment-derived eukaryotic DNA viruses, underrepresentation of Pepper mild mottle virus (RNA virus) and multiple bacteriophage lineages (DNA viruses). 10 virus species including 1 RNA virus, 9 DNA virus and pepper chlorotic spot virus were inversely correlated with COVID-19 severity	Zuo et al., 2021b
15 COVID-19 patients, 6 community acquired pneumonia patients, 15 healthy controls	Virome	Hong Kong, China	Gut DNA virome diversity was decreased. The fecal DNA virome of COVID-19 patients was mainly composed of crAss-like phages, *Myoviridae, Siphoviridae, Guaphage, Podoviridae*, and *Microviridae.* (the metagenomic data were obtained from a study of Zuo et al., 2020b, available publicly at the National Center for Biotechnology Information Sequence Read. Archive BioProject accession number PRJNA624223)	Lu et al., 2021

### Composition and changes in the respiratory microbiota

As the main target organ for the SARS-CoV-2 attack, the respiratory microbiota is also affected by COVID-19. Specimen collection is an important part when studying respiratory microbiota. Upper respiratory tract samples include throat and nasal swabs, while lower respiratory tract samples include deep cough sputum, bronchoalveolar lavage fluid, respiratory tract extracts, etc. Oropharyngeal and nasopharyngeal sites are those selected primarily for many studies because of the convenience of drawing samples from the upper respiratory tract. By comparing *16S rRNA* sequencing results of nasopharyngeal samples from 59 adults, including COVID-19 patients and healthy controls, Rosas-Salazar et al. (2021) found that *Peptoniphilus lacrimalis, Campylobacter hominis*, and *Prevotella 9 copri* were more abundant in patients with SARS-CoV-2 infection and in those with a high viral load, whereas in those without SARS-CoV-2 infection and those with a low viral load during COVID-19, *Corynebacterium* unclassified, *Staphylococcus haemolyticus* and *Prevotella disiens were enriched*. By metagenome sequencing of nasopharyngeal microbiota, researchers revealed a decrease in microbiota diversity in patients with confirmed COVID-19 and described microbial differential changes compared with SARS-CoV-2-negative patients (Mostafa et al., 2020). Similar to gut microbial changes, oropharyngeal changes in confirmed patients with COVID-19 (Gao et al., 2021) showed a decrease in butyrate-producing bacteria and an increase in opportunistic pathogens. *Firmicutes* increased in COVID-19 patients, while *Bacteroidetes, Proteobacteria*, and *Patellobacterium* decreased compared to healthy controls at the phylum level. At the genus level, among a total of 62 distinguished genera, 53 genera, including *Neisseria, Alloprevotella*, and *Prevotella*, were significantly reduced in patients with COVID-19, while nine genera, including *Streptococcus* and *Granulicatella* were increased. Metagenome sequencing of oropharyngeal swab samples from COVID-19 patients (Ma et al., 2021) (including mild, moderate, severe, and critical cases), 29 influenza patients, and 28 healthy controls revealed unique oropharyngeal microbiota characteristics of COVID-19 patients: opportunistic pathogens such as *Veillonella, Megacoccus* were enriched, while *Pseudopropionibacterium, Streptococcus, Rothella* were consumed. Furthermore, alteration of the oropharyngeal microbiota was associated with the severity of COVID-19, *Streptococcus sp.* and *Peptoniphilus sp.* were negatively correlated with the severity of COVID-19. Conversely, *Klebsiella sp., Acinetobacter sp.*, and *Serratia sp.* were positively correlated with the severity of COVID-19. The small circular DNA viruses *Anelloviridae* and *Redondoviridae* (which were shown to be enriched in respiratory samples from critically ill patients, Abbas et al., 2019) in oropharyngeal samples were positively correlated with intubation during hospitalization, as well as higher WHO scores (Ma et al., 2021). There has also been evidence of oropharyngeal microbial disorders associated with disease severity, and the possibility of assessing disease severity by microbiota signatures at early time points has been proposed (Merenstein et al., 2021). The duration of the intensive care unit and the type of oxygen support, as well as related treatment methods such as the use of antibiotics, can affect the composition of the upper respiratory tract microbiome, and subsequent studies may be needed to control the influence of these confounders (Llorens-Rico et al., 2021). In addition to changes in microbiota composition, the microbiota functions of COVID-19 patients have also changed. Compared with the control and influenza groups, the oropharyngeal microbiota of COVID-19 patients was enriched in exogenous biodegradation and metabolism, preferentially metabolizing specific amino acids such as tyrosine and phenylalanine (Ma et al., 2021). Few studies of lower respiratory tract microbiota are available in hospitalized patients with mild to moderate COVID-19 due to sampling difficulties. Thus, researchers have analyzed the bronchoalveolar lavage fluid (BALF) and found that the lung microbiota composition of patients with COVID-19 and community-acquired pneumonia was significantly different from healthy individuals, but did not specifically describe the difference (Shen et al., 2020). Identifying the microbiota of the lower respiratory tract can further aid treatment and prevent the progression of the disease to severe lung infections and respiratory failure.

Although most patients with COVID-19 currently have mild symptoms, there are a significant number of critically ill patients and even deaths. Numerous studies have confirmed that patients with severe COVID-19 have unique respiratory microecological characteristics. The diversity of oropharyngeal microorganisms in patients with COVID-19 is significantly reduced, and antibiotic resistance genes are increased, which is more evident in critically ill patients (Ma et al., 2021). The main respiratory microbiota in severely ill COVID-19 patients was *Staphylococcus epidermidis*, *Burkholderia cepacia complex* (BCC), or *Mycoplasma spp.* (including *M. hominis and M. orale*). Respiratory samples in mild and severe cases were tested at the same time to minimize errors, and Staphylococcal RNA was not detected in respiratory samples in all mild cases. Thus, *Staphylococcus* is difficult to consider as a contamination during sampling or detection, and may be characteristic of the gut microbiota in severe patients (Zhong et al., 2021). Turning to the lower respiratory tract, Merenstein et al. (2021) also confirmed that critically intubated COVID-19 had low microbiota diversity in the lower respiratory tract and had a predilection for *Staphylococcus*. Another study analyzed BALF from COVID-19 patients and healthy controls and concluded that bacterial diversity in the lower respiratory tract of COVID-19 patients was significantly higher than that of healthy controls (Han et al., 2022). This contradictory conclusion may be due to the BALF data from the COVID-19 and control group were obtained from multiple independent studies and the severity classification of the patients was unclear. Severely ill patients with COVID-19 often present with respiratory distress and hypoxemia, which require high-flow oxygen or mechanical ventilation (Meng et al., 2020). Tracheal intubation allows bacteria to easily enter the lower respiratory tract and colonizes the trachea (Craven and Hjalmarson, 2010; de Carvalho Baptista et al., 2018). Some oral microbiomes are more abundant in mechanically ventilated COVID-19 patients than in non-mechanically ventilated COVID-19 patients, further validating this notion (Llorens-Rico et al., 2021). Remarkably severe patients have a poor ability to cough and expel sputum spontaneously, leading to microorganisms accumulating in the alveoli and tracheobronchial tubes, which can easily lead to disturbance of the pulmonary microecosystem and increase the risk of co-infection and (or) secondary infection. Respiratory viral infection causes changes in microbiota composition and function, disrupts the immune barrier function of commensal bacteria, and is prone to cause co-infection and (or) secondary infection. Changes in the microbiome may also alter the regulation of infection by immune cells (Hanada et al., 2018; Manohar et al., 2020; Liu Y. et al., 2022). There have been many reports of COVID-19 co-infections and secondary infection (Bao et al., 2020; Chen et al., 2020; Cuadrado-Payán et al., 2020; Lansbury et al., 2020; Li J. et al., 2020). Patients with COVID-19, especially those who are critically ill, are more prone to co-infection and (or) secondary infection. Exploring the complex connection between the microbiota and the host immune system will provide a basis for the management of the health of critically ill patients and the application of antibiotics.

The respiratory tract microorganisms identified in patients with COVID-19 mentioned above usually refer to adult patients. Xu J. et al. (2021) pioneered the study of the dysbiosis of the upper respiratory tract of children with COVID-19. The most important clinically relevant findings were that the *Comamonadaceae* increased significantly in the upper respiratory tract, and the pathogenic bacterium *Pseudomonas* persists and dominates the microbiota in both the upper respiratory tract and the gut. The respiratory microbiota in children gradually deteriorates during treatment, and persistent dysbiosis may cause short-term and long-term health problems. This study may provide ideas for microbiota interventions for children during COVID-19 outbreaks.

### Composition and changes in the oral microbiota

The oral microbiota is a significant source for the lung microbiota in healthy humans (Bassis et al., 2015). Based on specific anatomical locations, the balance of the oral microbiome is critical to oral and systemic health (Gao et al., 2018). The impact of oral microbiota on COVID-19 has been evaluated. Oral microbial diversity was observed in COVID-19 patients, *Prevotella salivarius* and *Veillonella infantis* were unique in COVID-19 patients, whilst *Neisseria perflava* and *Rothia mucilaginosa* were distinct in controls (Iebba et al., 2021). Previous research confirmed that (Wu Y. et al., 2021) compared to controls, *Neisseria, Corynebacterium*, and *Actinobacillus* at the genus level, as well as *P. intermedia* and *T. amylovorum* were significantly depleted in COVID-19 patients. On the contrary *Veillonella, Granulicatella*, and *Campylobacter* at the genus level, as well as *R. mucilaginosa, H. parainfluenzae* were increased in abundance. Pathways associated with the TCA cycle in the oral microbiota of COVID-19 patients were inhibited. Recently Ren et al. (2021) found a decrease in oral butyrate-producing bacteria (*Porphyromonas* and *Fusobacterium*) and an increase in lipopolysaccharide-producing bacteria (*Leptotrichia* and *Selenomonas*) and established a non-invasive diagnostic model based on oral microbial characteristics. A recent study has linked oral microbiota dysbiosis to the duration of long COVID symptoms and disease outcomes and demonstrated the members of the genera *Prevotella* and *Veillonella*, which are inflammation-inducing and LPS-producing microbiomes, were more abundant in COVID-19 patients with prolonged symptoms (Haran et al., 2021). There is a potential link between oral microbiota dysbiosis and bacterial co-infection in patients with COVID-19 (Bao et al., 2020). Recognizing oral microbiota perturbations may help to deepen our understanding of the mechanisms of virus-bacteria interaction in COVID-19 and to undertake protective oral hygiene measures to reduce this infection.

## Microbiota dysbiosis, SARS-CoV-2 infection and host immunity

SARS-CoV-2 can activate innate immunity as well as adaptive immunity. Research on the imbalanced host response to SARS-CoV-2 has included cell lines, ferrets, and COVID-19 patients, which were used to analyze the transcriptional response of SARS-CoV-2. Blanco and colleagues determined that viral infection could suppress host type I and type III interferon (IFN) expression (Blanco-Melo et al., 2020). This finding also agrees with the observations of Smith, which showed that nasopharyngeal viral load was inverse to the response to IFN (Smith et al., 2021). Type I IFN responses (characterized by low IFN-α and no IFN-β production and activity) are severely impaired in critically ill patients with COVID-19, resulting in exacerbated inflammatory responses and persistent blood viral loads (Hadjadj et al., 2020). Antiviral responses, which are mediated by IFN, are central to host defense against viral infection. Both type I and type III IFNs signal through the JAK-STAT pathway to activate the transcription factor complex ISGF3, which encodes a protein that acts through multiple mechanisms to limit viral infection (Lazear et al., 2019; Mesev et al., 2019). SARS-CoV-2 inhibits the JAK-STAT pathway in infected cells and interferes with IFN-mediated signaling (Triana et al., 2021).

Cytokines are small molecular proteins secreted by immune cells for intercellular signal transduction when pathogens invade cells, including interferon (IFN), interleukin (IL), tumor necrosis factor (TNF), etc. (Tisoncik et al., 2012). Under normal circumstances, pro-inflammatory (such as IL1β, IL6, IL12, TNF, and IFN-γ) and anti-inflammatory cytokines (such as IL4, IL10, IL13, and TGF-β) maintain a dynamic balance and regulate the inflammatory response. Notably, when the immune response is excessively activated, pro-inflammatory cytokines are produced in large quantities in a brief period. They will attack the body’s own cells, resulting in a cytokine storm, which is a significant cause of acute respiratory distress syndrome, septic shock, and multiple organ failure. From SARS-CoV (Huang et al., 2005), ebola (Younan et al., 2017), and SARS-CoV-2 (Ramasamy and Subbian, 2021), cytokine storms have been frequently mentioned. The cytokine storm is essential to transform patients with COVID-19 from mild to severe and critical, as well as an important cause of death in severe and critical patients (Tang et al., 2020b). A clinical study of COVID-19 patients in Wuhan found that plasma levels of TNFα, IL2, IL7, IL10, IP10, MCP1, MIP1A, and GSCF in patients in the intensive unit (ICU) were higher than in non-ICU patients (Huang et al., 2020). Higher levels of pro-inflammatory macrophages, neutrophils, and inflammatory cytokines, especially interleukin (IL)-6, IL-8, and IL-1β were conducted in the BALF of patients with severe/critical COVID-19 (Liao et al., 2020). A review explains the possible mechanism of actions among SARS-CoV-2, microbiota, and cytokine storm (Liu Y. et al., 2022): (i) severe respiratory virus infection, the immune response excessive activate, leading to cytokine storm; (ii) SARS-CoV-2 infection leads to the destruction of the intestinal barrier, and bacterial lipopolysaccharide stimulates the production of cytokines; (iii) SARS-CoV-2 infection leads to the growth of opportunistic pathogens and bacterial secondary infection, leading to cytokine storm through a series of immune effects; (iv) viruses interfere the gut microbiota to promote the production of harmful metabolites and stimulate cytokines.

The intestinal tract is the largest immune organ in the human body. A healthy intestinal microbiota is necessary to maintain immune homeostasis of the body. Microbiota dysbiosis caused by SARS-CoV-2 infection will interfere with host immunity. It has been shown that the opportunistic pathogen *B. Contaminans* (the main microorganism in the respiratory tract of critically ill COVID-19 patients) was associated with higher levels of circulating hypersensitive C-reactive protein (hs-CRP) and IL-6 and lower total lymphocyte counts, CD3 + T, and CD4 + T counts. Furthermore, IL-6, IL-8, and hypersensitive-CRP circulating levels were positively correlated with these virulence genes associated with bacterial invasive capacity (Zhong et al., 2021; Sun et al., 2022). Further studies have shown that the imbalance of nasopharyngeal flora in patients with COVID-19 affects local and systemic cytokines and antibody. For instance, microbial α-diversity and Corynebacterium were negatively correlated with CCL2, whereas Staphylococcus was positively correlated with inflammatory cytokines (IL-6 and TNF) (Smith et al., 2021).

The researchers further investigated the mechanisms underlying the impact of the microbiota on host immunity. Butyric-producing bacteria have been validated to promote IL22 production to maintain intestinal homeostasis (Yang W. et al., 2020) and downregulate genes associated with SARS-CoV-2 infection (Li J. et al., 2021). Short-chain fatty acids promote CD8 + T cell function to facilitate the clearance of the influenza virus (Trompette et al., 2018). In addition, they can also bind to GPR109A, a butyrate receptor, that induces differentiation of Treg cells and IL-10-producing T cells to exert an anti-inflammatory effect (Singh et al., 2014). However, the short-chain fatty acid-producing microbiota is inversely correlated with the fecal abundance of SARS-CoV-2 (Zuo et al., 2021a). A previous study proposed that the *Bacteroidetes* phylum, which is reduced in severe and critically ill patients with COVID-19, could activate colonic dendritic cells through the TLR4-TRIF pathway using its bacterial outer membrane glycolipids and promote dendritic cells to secrete IFNβ to enhance host resistance against viral infection (Stefan et al., 2020). *Lactobacillus*, which was known to be significantly reduced in COVID-19 patients (Tang et al., 2020a), can produce the aryl hydrocarbon receptor (AhR) ligand-indole-3-aldehyde that promotes AhR-dependent IL22 transcription and improves resistance to mucosal inflammation (Zelante et al., 2013). *Veillonella*, an opportunistic pathogen enriched in COVID-19, was found to lead to Th17 cell recruitment, neutrophil enrichment, and IL7 inflammatory phenotype activation (Tsay et al., 2021). Taken together, the gut microbiota can regulate the immune response through translocation of bacterial products or induction of anti-inflammatory cytokines (Sarkar et al., 2021). Microbiota in IBD patients can alter disease susceptibility through distinct immune pathways, suggesting specific therapies targeting different microbial signatures (Roy et al., 2017). Hence, it could conceivably be hypothesized that treat COVID-19 by targeting the microbial community.

The gut microbiome may alter susceptibility to SARS-CoV-2 (Dhar and Mohanty, 2020), and in turn, SARS-CoV-2 infection can cause an imbalance in the microbiota. SARS-CoV-2 infects intestinal epithelial cells and damages the integrity of the intestinal barrier (Guo Y. et al., 2021). Transcriptional analysis of biomimetic gut-on-chip shows that virus infection causes epithelial cells to produce abnormal proteins and RNA metabolism to activate immune responses (Guo Y. et al., 2021). Additionally, an altered intestinal barrier leads to changes in the intestinal microbiota and its metabolites, translocation of bacteria into the blood circulation or other sites, causing aggravation of systemic or local inflammation followed by damage to multiorgan function (Fernandes et al., 2019; Lau et al., 2022; Martel et al., 2022). Together, these studies provide important insights into Interactions among microbiota, SARS-CoV-2 and host immunity.

## Potential mechanisms for the impact of microbiota on COVID-19

### Microbiota and angiotensin converting enzyme 2

Previous research on ACE2 has been relatively mature. ACE2, a vital regulatory protein of the renin-angiotensin system, can change Angiotensin II into Ang (1-7) (Kuba et al., 2010). ACE2, Ang (1-7), and its receptor Mas form an anti-inflammatory and antioxidant axis to maintain systemic metabolic homeostasis (Simoes e Silva et al., 2013; Santos et al., 2018). After the outbreak of the COVID-19 epidemic, it was found that ACE2 mediates the entry of SARS-CoV-2 into the human body (Zhou P. et al., 2020). SARS-CoV-2 relies on transmembrane serine protease 2 (TMPRSS2) and furin to cleave and activate the SARS-CoV-2 envelope spike protein (S protein) and the binding of the S protein to its receptor ACE2 to infect host cells (Heurich et al., 2014; Bestle et al., 2020; Hoffmann et al., 2020; Xu X. et al., 2020). ACE2 and TMPRSS2 are widely distributed in ileocolonic epithelial cells, salivary glands, and oral mucosa epithelial cells (Guo M. et al., 2021; Huang N. et al., 2021).

ACE2 stabilizes the expression of amino acid transporter B0AT1, controls the uptake of neutral amino acids, regulates the expression of antimicrobial peptides (AMP), and affects the microbial composition, all independently of the renin-angiotensin system (RAS) (Hashimoto et al., 2012; Viana et al., 2020). But ACE2 affects the microbial composition through mTOR-mediated synthesis of AMPs (Perlot and Penninger, 2013). SARS-CoV-2 infection negatively regulates cellular ACE2 expression and inhibits its enzymatic activity (Trottein and Sokol, 2020; Triana et al., 2021; Li et al., 2022), leading to disruption of the intestinal barrier, microbiota dysbiosis, and worsening of systemic inflammation (Hashimoto et al., 2012; Penninger et al., 2021). In turn, the gut microbiota can also regulate the ACE2 expression in the intestine tract. A notable example is that microbiota colonized conventionalized GF (GFC) rats have lower colonic ACE2 expression and higher levels of tryptophan metabolites, hydroxy kynurenine, and kynurenic acid compared with germ-free rats (Yang T. et al., 2020). Subsequent studies by Edwinson et al. (2021) further confirmed this view. Transplantation of irritable bowel syndrome (IBS) and healthy human fecal microbiota into germ-free mice, after microbial colonization, marked downregulation of ACE2 expression has been observed both in healthy microbiota and IBS microbiota humanized mice (Edwinson et al., 2021). *Bacteroidetes*, including *Bacteroides dorei* and *Bacteroides thetaiotaomicron* were significantly negatively correlated with the SARS-CoV-2 load in feces. Meanwhile, it can downregulate ACE2 expression in the gut. In contrast, Firmicutes, positively correlated with COVID-19 severity, can up-regulate ACE2 expression (Zuo et al., 2020b). This suggests a paradoxical result that ACE2 may be upregulated in severely ill patients with COVID-19 and those with a high viral load. Researchers proffered that the expression of ACE2 in the respiratory epithelium of COVID-19 patients increased three times compared with the control group (Chua et al., 2020). This contradictory result may be due to the dual role of ACE2 (Yan et al., 2020). On the one hand, as a receptor for SARS-CoV-2, ACE2 facilitates the invasion of cells by the virus. On the other hand, as a negative regulator of the renin-angiotensin system and an amino acid transport regulator, ACE2 protects the body from damage under various pathological conditions (Viana et al., 2020; Yan et al., 2020). For instance, ACE2 plays a key role in protecting mice from severe acute lung injury caused by acid inhalation or sepsis (Imai et al., 2005). Hence, in-depth research on the complex relationship between ACE2 and microorganisms is still warranted.

### Relationship between microbial metabolites and COVID-19

Numerous studies have illustrated that the microbiota can participate in disease progression and affect the host via various pathways through their metabolites, such as short-chain fatty acids, aromatic compounds, amino acids, bile acids, vitamins, and lipids (McCarville et al., 2020; Van Treuren and Dodd, 2020). Reviewing the literature, substantial data is available on the association between microbial metabolites and several diseases. One study demonstrated that mice with high levels of circulating short-chain fatty acids (SCFA) had reduced susceptibility to allergic inflammation in the lungs (Trompette et al., 2014). Knockout of the gastrointestinal commensal bacterial bile acid metabolism pathway in mice reduced the proportion of colonic RORγ + regulatory T cells and improved the susceptibility of the colon to colitis (Song et al., 2020). The microbial product indole stimulates the expression of PFKFB3, an essential regulatory gene associated with glycolysis, and inhibits macrophage activation against non-alcoholic fatty liver disease (Ma et al., 2020).

**FIGURE 1:**
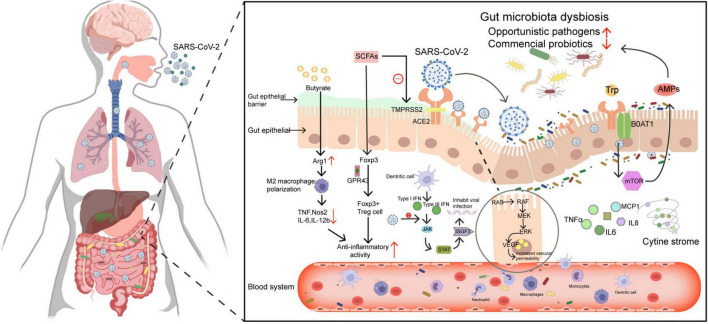
Schematic diagram of the relationships among SARS-CoV-2, ACE2, host immunity, and microorganisms. (1) Microbiota metabolites such as butyrate exert anti-inflammatory effects by up-regulating arginase 1 (Arg1) expression, down-regulating Nos2, IL6, and IL-12, and inhibiting tumor necrosis factor (TNF) activity. In addition, short-chain fatty acids inhibit histone deacetylases and increase the expression of foxp3 through the GPR43 receptor, thereby enhancing the regulatory function of FOXP3 + Treg cells resulting in anti-inflammatory effects. On the other aspect, short-chain fatty acids can inhibit TMPRSS2 gene expression and up-regulate antiviral pathways to inhibit viral entry. (2) The SARS-CoV-2 is activated by TMPRSS2, binds to ACE2 and enters the gut, can destroy the gut barrier and causes microbiota dysbiosis. (3) SARS-CoV-2 infection suppresses the JAK-STAT pathway of type I and type III interferon responses, and the protein encoded by ISGF3 that limits viral infection will be diminished. Besides, SARS-CoV-2 infection downregulates the expression of ACE2, weakens its ability to regulate the RAS system, and over activates the immune response. Dysregulated gut microbes and their metabolites can also stimulate the production of cytokines, causing a cytokine storm. (4) SARS-CoV-2 spike protein activates the Ras-Raf-MEK-ERK-VEGF pathway in intestinal epithelial cells and promotes vascular endothelial growth factor (VEGF) production, which leads to vascular permeability and inflammation. (5) ACE2 regulates the expression of the amino acid transporter B0AT1, which affects microbiota composition through mTOR-mediated antimicrobial peptide production.

The microbiota interferes with SARS-CoV-2 infections not only through the microecosystem but also through its complex and diverse microbiota metabolites. A study proposed that the increase of inflammatory mediators such as TNFSF14 and the oncostatin M in plasma from COVID-19 patients was related to the increase in bacterial products in plasma (Arunachalam et al., 2020). It is now well established from various studies that the abundance of short-chain fatty acid-producing bacteria in COVID-19 patients was reduced (Gu et al., 2020; Zuo et al., 2021a). Recently, to study the relationship between gut microbiota metabolomics and the pathogenesis and severity of COVID-19, Zhang et al. (2022) also found that impaired synthesis of SCFA and L-isoleucine in the gut microbiome of patients with COVID-19, which is also associated with increased disease severity and inflammatory markers such as CRP and CXCL-10, increased urea synthesis as well. SCFA induce sophisticated effects, including anti-inflammatory, immune regulation (Park et al., 2015; Garrett, 2020), and metabolism (Cani, 2018). For example, it exerts anti-inflammatory effects by regulating the functions of immune cells such as macrophages and Treg cells (Rooks and Garrett, 2016; Scott et al., 2018). Butyrate is a short-chain fatty acid that has been intensively studied. Interestingly, a recent systematic literature review concluded that butyrate inhibits SARS-CoV-2 infectivity by reducing the expression the of ACE2 and TMPRSS2 genes, upregulating the level of ADAM17, a metallopeptidase involved in ACE2 shedding, and upregulating multiple critical antiviral pathways such as TLR (Li J. et al., 2021). Three bacterial metabolites 5-hydroxytryptamine receptor agonist tryptamine, pyrazine 2,5-bis (3-indolylmethyl) pyrazine and N6-(D2-isopentenyl) adenosine, exert anti-SARS-CoV-2 activity and share the similar structures and functions to clinical antiviral drugs (Piscotta et al., 2021). High doses of 25-hydroxyvitamin D notably reduced ICU admission for hospitalized patients with COVID-19 (Entrenas Castillo et al., 2020). These findings provide some support for the conceptual premise that targeting the microbiota and its metabolites may be useful for the treatment of COVID-19. The relationships among SARS-CoV-2, ACE2, host immunity, and microorganisms are shown in Figure 1.

## Microbiota may serve as a potential diagnostic marker

The utility of microbiota signatures for the non-invasive diagnosis of liver cirrhosis (Oh et al., 2020), colorectal cancer (Coker et al., 2022), etc., has attracted attention, prompting us to consider the potential role of the microbiota in the diagnosis of COVID-19. The current gold standard for diagnosing COVID-19 is PCR testing. However, due to virus mutation (Food and Drug Administration, 2021^1^), different sampling sites, sampling time points (He et al., 2020), or detection methods (Pan Y. et al., 2020), detection efficiency is reduced and false negatives may occur. Therefore, an efficient and accurate diagnostic method is urgently required. Gu first proposed five specific gut microbiomes as a diagnostic marker to distinguish COVID-19 from healthy controls, with a ROC-plot AUC value of 0.89 (Gu et al., 2020). Nevertheless, Klann et al. (2021) argued for the practicality of this diagnostic method, arguing that observed differential microbes may be affected by sequencing efficiency or diarrhea symptom. Gut microbes should be more of a potential therapeutic target rather than a diagnostic biomarker (Klann et al., 2021). Subsequently, Ren et al. (2021) analyzed the oral microbiome of 48 confirmed patients and 100 healthy controls to identify markers that could differentiate microorganisms in confirmed patients and healthy people, and then established an oral microbial diagnostic model. The diagnostic model was validated in a validation cohort (24 confirmed patients and 50 healthy controls) and showed high diagnostic efficacy with an AUC value of 95.75% between the two groups. In addition, the diagnostic model obtained from tongue-coating samples in Henan province was applied to 74 confirmed patients in Hangzhou in order to achieve cross-regional validation, with an AUC value of 87.24% compared with healthy controls. The highlight of the study was that this approach could increase the diagnosis rate of suspected patients, that is, patients with negative RT-PCR but positive IgG antibody accompanied by clinical symptoms and imaging manifestations (Ren et al., 2021). Hence, it could possibly be hypothesized that the microbiota can be used as an important non-invasive auxiliary diagnosis to reduce the false-negative rate and the occurrence of missed diagnoses. Other studies have established and validated diagnostic models using the oropharyngeal (Gao et al., 2021) and gut microbiota (Li S. et al., 2021). Table 2 shows studies on microbiota as a model for non-invasive diagnosis of COVID-19. These findings, while preliminary, indicate the possibility of microbial markers in the future diagnosis of COVID-19. However, these results should be interpreted with caution. The current microbial diagnostic markers for diseases are only in the preliminary research stage, and there are many shortcomings in using them as an independent diagnostic method. First, the specificity of the microbiota as a diagnostic marker is insufficient. A recent study showed overlap of microbiota signatures in inflammatory bowel disease and type 2 diabetes, for example, decreased levels of *Faecalibacterium*, *Roseburia*, and *Collinsella* (Metwaly et al., 2022). Surprisingly, these changes have also been observed in COVID-19 (Moreira-Rosário et al., 2021; Reinold et al., 2021). Second, the complexity of diseases and the heterogeneity of the microbiota pose considerable challenges to microbial research. Current research can only distinguish COVID-19 patients from healthy individuals by microbiota signatures, but not other diseases. Due to the complexity of microbial ecology, experiments and validation are required in larger cohorts to determine the optimal microbial profiles.

**TABLE 2:** Microbial markers as a diagnostic model for COVID-19.

Study cohort	Characterized microbiota	Diagnostic efficacy	Ref.
Gut microbiota 30 COVID-19 patients, 24influenza A (H1N1) patients, 30 matched healthy controls (HC)	*Fusicatenibacter, Romboutsia, Intestinibacter, Actinomyces, Erysipelatoclostridium*	89% (95% CI, 80%–97%)	Gu et al., 2020
Gut microbiota Discovery cohort (37 confirmed patients, 10 healthy controls)	*Eubacterium hallii, Coprococcus catus, Bacteroides vulgatus, Prevotella bivia* etc., a total of 15 species	93.3% (95% CI of 79.8–100.0%, *p* < 0.001) in cross-regional validation of Changsha:10 COVID-19 patients	Li S. et al., 2021
Gut microbiota Discovery cohort (24 confirmed patients, 48 healthy controls)	OTU1741 (*Halomonas*), OTU1314 (*Pelagibacterium*), OTU1473 (*Faecalibacterium*), OTU1000 (*Blautia*), etc., a total of 7 OTUs	99.31% (95% CI 97.66%-100%, *p* < 0.0001) in validation cohort:12 confirmed patients, 24 healthy controls	Ren et al., 2021
Oropharyngeal microbiota Discovery cohort (48 confirmed patients, 94 healthy controls)	OTU10 (*Alloprevotella*), OTU26 (*Prevotella*), OTU34 (*Halomonas*), OTU5 (*Haemophilus*), etc., a total of 8 OTUs	99.3% (95% CI 98.21%-100%, *p* < 0.0001) in validation cohort:25 confirmed patients, 46 healthy controls	Gao et al., 2021
Oral microbiota Discovery cohort of Henan (48 confirmed patients, 100 healthy controls)	OTU1642 (*Haemophilus*), OTU1277 (*Actinomyces*), OTU1868 (*Prevotella*), OTU648 (*Oribacterium*), OTU1454 (*Fusobacterium*), etc., a total of 8 OTUs	95.75% (95% CI 90.99%-100%, *p* < 0.0001) in validation cohort: 24 confirmed patients, 50 healthy controls *p* < 87.24% (95% CI 80.5% to 93.98%, *p* < 0.0001) in cross-regional validation of Hangzhou:74 confirmed patients	Ren et al., 2021

## Microbiota: an influencing factor and predictor of COVID-19 prognosis

During the more than 2 years of the COVID-19 epidemic, due to the continuous mutation of the virus, the constant evolution of infectivity, and immune evasion, the number of infected people around the world has continued to grow. With the in-depth study of COVID-19, the constant maturation of treatment methods, and preventive vaccination strategies, the number of survivors of COVID-19 has increased significantly compared to the initial outbreak period. However, an increasing number of individuals pay closer attention to the sequelae of COVID-19, and the concept of Long COVID was proposed (Rubin, 2020; Callard and Perego, 2021), namely, symptoms that persist after recovery from COVID-19. Long COVID affects multiple systems and has many adverse effects on the physical and mental health (Del Rio et al., 2020; Al-Aly et al., 2021) of survivors. The sequelae of COVID-19 have been reported, including fatigue, muscle weakness, sleep disorders (Huang C. et al., 2021), cognitive deficits (Hampshire et al., 2021), sexual dysfunction (Sansone et al., 2021), alopecia (Xiong et al., 2021), loss of taste, loss of smell, and other sequelae. Strikingly, the proportion of patients with sequelae in different countries has been reported to be at least 50% (Nalbandian et al., 2021).

Given the critical role that the microbiota plays in the pathogenesis and clinical manifestations of COVID-19, Huang and colleagues conducted a follow-up study including 106 patients with COVID-19 for up to 6 months (Liu Q. et al., 2022) to investigate the gut microbiota diversification of patients with post-acute COVID-19 syndrome (PACS). Surprisingly, it was discovered that the composition of the gut microbiota at admission was related to PACS. Patients with PACS had lower Shannon diversity and gut microbiota richness compared to those without PACS and without COVID-19. Despite changes in bacterial species upon admission, the gut microbiota of patients without PACS recovered to basically the same level as non-COVID-19 controls at 6 months, while those with PACS exhibited a unique gut microbiota composition, characterized by a higher abundance of *Bacteroides vulgatus* and *Ruminococcus gnavus*, and a lower abundance of *F. prausnitzii* and *Collinsella aerofaciens*. Furthermore, in this study, microbiota patterns associated with neuropsychiatric symptoms, respiratory symptoms, and hair loss were identified. Stable gut microbiota composition is associated with a better prognosis for COVID-19. In addition to being a non-invasive diagnostic marker, gut microbiota signatures can also predict death in patients with COVID-19 during hospitalization (Schult et al., 2022), as an indicator of prognostic.

In addition to long-term sequelae, recurrence after recovery cannot be ignored. Taking the Ebola virus disease as an example (Keita et al., 2021), there was a further outbreak in Africa from February to June 2021, and sequencing of the viral genes showed that it was the same as the virus responsible for the outbreak 7 years prior, and it was unlikely to be of animal origin. Epidemiological investigation validated this time that patient No. 0 was the rehabilitation 7 years ago, suggesting that the virus may remain in a low-replication state and persist. Similarly, recurrence of COVID-19 patients also occurred after cure. In one study, 173 patients recovering from COVID-19 who were discharged from the hospital were monitored for at least 1 month, and 12 patients were re-detectable positive (positive for SARS-CoV-2 RNA in throat swabs). The diversity and composition of the fecal flora of patients with re-detect positive (RP) were different from those of non-RP, and the proportion of positive fecal SARS-CoV-2 RNA on the day of discharge was significantly higher (60% vs. 11.3%), suggesting that the intestinal may act as a reservoir for SARS-CoV-2 leading to RP. Remarkably, all eight patients with *Prevotella*-enriched microbiota were non-RP (Tao et al., 2021). However, with a small sample size, caution must be applied, as the findings may not be accurate. Anyway, these studies raised the possibility that strengthening the long-term management of recovered patients seems essential and that the microbiome may be helpful for health management as a criterion for the early identification of prognosis or relapse.

## Application prospect of microbiota in the treatment of COVID-19

### Probiotics and prebiotics

Probiotics are non-pathogenic microorganisms that, when taken in appropriate doses can improve the microecological balance of the host and are beneficial to host health. Prebiotics are substances that cannot be digested by the host but can be selectively utilized by host microorganisms and converted into substances valuable to the host’s health (Gibson et al., 2017). Probiotics and prebiotics play an important role in improving the intestinal barrier, interacting with the host and microbiota, generating antimicrobial products and organic acids, resisting pathogens, regulating immunity, and affecting metabolism, meanwhile can be used for adjuvant therapy of various diseases (Sanders et al., 2019), guidelines for the use of probiotics in children already exist (Cameron et al., 2017). As mentioned above, the microbiota of patients with COVID-19 has undergone significant changes, and most of these changes are unfavorable for the body. Improving intestinal microecology is expected to become an adjuvant treatment for COVID-19 (Hu et al., 2021), thus improving the body’s immunity and promoting recovery. A bibliometric analysis of 84 scientific studies on microecological interventions with COVID-19 showed that probiotics or prebiotics could enhance resistance to COVID-19 infection, reduce disease duration and symptoms by strengthening the mucosal barrier and modulating the host immune system, as well as strengthening the gut-lung axis (Xavier-Santos et al., 2022). The effect of probiotics on the treatment of COVID-19 has also been widely explored. Oral probiotics relieved symptoms such as diarrhea, fatigue, and fever, and, in addition, reduced the proportion of admissions to the ICU (d’Ettorre et al., 2020), and notably diminished the risk of death in hospitalized patients (Ceccarelli et al., 2020). A randomized controlled trial conducted in Mexico confirmed that the probiotic group had a higher complete remission rate and shorter duration of gastrointestinal and parenteral symptoms, viral load of nasopharyngeal, and lung infiltrates compared to the placebo group. Specific IgM and IgG antibodies to SARS-CoV-2 also increased significantly (Gutierrez-Castrellon et al., 2022). Contrary to expectations, another study did not find significant differences in mortality, hospital stay, levels of inflammatory biomarkers, or ICU admissions between probiotic-treated and control-treated patients. The probiotic just reduced the duration of diarrhea resolution and prevented hospital-acquired diarrhea when treated with a single antibiotic (Ivashkin et al., 2021). Regarding recovery after probiotic treatment, six out of the eight COVID-19 patients who received adjuvant probiotic treatment recovered fecal microbiota composition after 14 days of treatment, more similar with healthy controls (Wu C. et al., 2021).

The role of probiotics and prebiotics has been widely debated. Critically ill patients who receive probiotics have a lower risk of ventilator-associated pneumonia (VAP) (Manzanares et al., 2016). Lactobacillus rhamnosus GG prevented high-risk ICU populations from VAP (Morrow et al., 2010). However, there were no significant differences between the probiotic group (patients who received Lactobacillus rhamnosus GG) and the placebo group in the prevention of VAP among critically ill patients, as confirmed by a randomized controlled trial (Johnstone et al., 2021). An unanticipated finding was that the use of probiotics might even increase the risk of bacteremia in ICU patients (Yelin et al., 2019). Given the conflicting results between different studies, more trials are needed for further verification, focusing on its rational use and safety. The application of probiotics in the clinical treatment of COVID-19 still requires long-term research and is not suitable for all patients (Mak et al., 2020). Nonetheless, at minimum, the following conditions must be met for successful application: bacteria that are beneficial to health, proper formulation, can tolerate colonization of gastric acid and bile in the gastrointestinal tract, and have been clinically tested to ensure safety and efficacy. Table 3 briefly summarizes several completed clinical trials of probiotics and symbiotics interventions and other undergoing clinical trials of microecological therapy.

**TABLE 3:** Several clinical studies of microecological intervention in COVID-19.

Identifier	Recruiting status	Study subjects	Age	Country	Sample size	Supplement	Intervention	Primary outcome	Access link
NCT04390477	Completed	Patients with a confirmed diagnosis of SARS-CoV-2 and require admission to the hospitalization	≥18 years	Spain	41	Probiotic	1 pill containing probiotic 1 × 10^∧^9 CFU or Placebo. 1 oral capsule per day for 30 days.	Percentage of patients with discharge to ICU	https://clinicaltrials.gov/ct2/show/NCT04390477
NCT04458519	Completed	COVID-19 patients not requiring hospitalization	18−59 years	Canada	23	Probiotic	Nasal irrigations with Probiorinse 2.4 Billion CFU (Colony-Forming Units) of *Lactococcus Lactis W136* or saline (NeilMed Sinus Rinse). Twice-daily for a period of 14 days	Change in severity of COVID-19 infection	https://clinicaltrials.gov/ct2/show/NCT04458519
NCT04507867	Completed	Stage III positive COVID-19 patients with comorbidities (type 2 DM, SAH, overweight/obesity with BMI < 35)	30−75 years	Mexico	80	Synbiotic	Combination of three B vitamins (B1, B6, and B12) “Neurobion” 10 mg solution for IM injection, One every 24 h for the first 5 days. Probiotics *Saccharomyces boulardii* CNCM I-745 “Floratil.” One morning and one evening 250 mg capsule during the first 6 days. One envelope of NSS-1 in the morning and one envelope in the afternoon mixed with 400 ml of water each, contain nutritional support system or placebo	Overall mortality at day 40 and overall survival	https://clinicaltrials.gov/ct2/show/study/NCT04507867
NCT04517422	Completed	Mild COVID-19 patients, cough, fever, dyspnoea, or headache, onset < = 7 days	18−60 years	Mexico	200	Probiotic	Probiotics (*Lactiplantibacillus plantarum CECT30292, Lactiplantibacillus plantarum CECT 7484, Lactiplantibacillus plantarum CECT 7485*, and *Pediococcus acidilactici CECT 7483*) or Placebo (maltodextrin). Oral for 30 days	Severity of COVID-19 progression; stay of patients at ICU and mortality ratio	https://clinicaltrials.gov/ct2/show/NCT04517422
NCT04621071	Completed	COVID-19 patients	≥18 years	Canada	17	Probiotic	Dietary Supplement: Probiotics (2 strains 10 × 10^∧^9 UFC) or Placebo (potato starch and magnesium stearate). From Day 1 to Day 10 two capsules a day, Day 11 to Day 25 one capsule a day, maximum of 25 days, if they are admitted to the hospital treatment will stop	Duration of symptoms of the COVID-19	https://clinicaltrials.gov/ct2/show/NCT04621071
NCT04734886	Completed	Previous diagnosis of COVID-19 (by positive PCR) or previous confirmation of seropositivity to SARS-CoV-2	18−60 years	Sweden	161	Probiotic	Dietary Supplement: *L. reuteri* DSM 17938 + vitamin D or Placebo + vitamin D. Two capsules per day for 6 months	SARS-CoV-2 specific IgG/IgM antibodies in serum between the study arms	https://clinicaltrials.gov/ct2/show/NCT04734886
NCT04824222	Not yet recruiting	COVID-19 patients with expected survival time, not taking into account SARS-CoV-2 infection, is at least 6 months. Hospitalization due to COVID 19 disease or disease with accompanied COVID 19.	≥18 years	Not provided	366	Fecal microbiota transplantation (FMT)	FMT be administered in double cover, gastro-resistant, enteric release capsules in 60 g dose or placebo	Incidence of adverse events after administration of IMP in the phase II/III, percentage of patients requiring escalation of oxygen therapy in non-invasive and invasive methods in phase III	https://clinicaltrials.gov/ct2/show/NCT04824222
NCT04847349	Completed	SARS-CoV-2 infection > 4 months prior confirmed	18−60 years	United States	54	Probiotic	Probiotic consortium OL-1, standard dose or Probiotic consortium OL-1, high dose or Placebo (potato starch, maltodextrin). A capsule once per day with breakfast for 21 days	Change in titer of serum anti-SARS-CoV-2 immunoglobulin G (IgG)	https://clinicaltrials.gov/ct2/show/NCT04847349
NCT04854941	Completed	COVID-19 patients	18−75 years	Russian Federation	200	Probiotic	Probiotics (10^∧^9 CFU of each strain: *Lactobacillus rhamnosus PDV 1705, Bifidobacterium bifidum PDV 0903, Bifidobacterium longum subsp. infantis PDV 1911* and *Bifidobacterium longum PDV 2301*) or standard regimen for COVID-19. Three times a day during the hospital stay but for no more than 14 days, the end point was day 14 or patient’s discharge or death	The number of died patients during hospitalization	https://clinicaltrials.gov/ct2/show/NCT04854941
NCT05043376	Completed	Hospitalized confirmed (RT-PCR) COVID-19 patients not receiving mechanical ventilatory support	≥18 years	Pakistan	50	Probiotic	Dietary Supplement: BLIS K12 (*Streptococcus salivarius K12*). Daily 2 oral BLIS K12 tablets for up to 14 days	Recovery and live discharge, number of patients with clinical improvement	https://clinicaltrials.gov/ct2/show/NCT05043376
NCT05175833	Completed	patients hospitalized in the ICU with severe acute respiratory syndrome	≥18 years	Brazil	70	Probiotic	Oral gel containing *streptococcus salivarius K12* and *lactobacillus brevis CD2* or containing placebo The oral gel was applied in the mouth every 8 h for 7 days	The occurrence of symptoms and signs of secondary bacterial pneumonia	https://clinicaltrials.gov/ct2/show/NCT05175833
IRCT20080826001096N8	Recruiting	Pregnant women with mild/moderate COVID-19	≥16 years	Iran	80	Synbiotic	Lactofem capsule, contains 500 mg of 4 strains of probiotic lactobacilli including *Lactobacillus acidophilus, Lactobacillus plantarum, Lactobacillus fermentum, Lactobacillus gasseri* and 38.5 mg of prebiotic substances including fructooligosaccharides or placebo Twice daily after meals for at least 7 days until hospitalization	Duration from the start of the study until the improvement of clinical symptoms.	https://trialsearch.who.int/Trial2.aspx?TrialID=IRCT20080826001096N8
IRCT20200117046164N2	Recruiting	Asthmatic children without positive history of COVID-19	6−18 years	Iran	90	Probiotic	Probiotic nasal spray contains *Lactobacillus reuteri and Lactobacillus casei* or rhinosaline nasal spray Administer twice daily, one puff in each nostril, for 1 month	Abnormality in smell, Axillary temperature above 38 Celsius degree, Development of respiratory and gasterointestinal symptoms	https://trialsearch.who.int/Trial2.aspx?TrialID=IRCT20200117046164N2
ChiCTR2000029999	Prospective registration	COVID-19 patients	≥18 years	China	60	Probiotic	Specific probiotic types not yet available	Several parameters associated to gut microbiome, fecal metabolomics, cytokines, biochemical, hematological, imageology, etc.	https://www.chictr.org.cn/historyversionpub.aspx?regno=ChiCTR2000029999

### Fecal microbiota transplantation

Research on probiotics has advanced further in the medical field; a typical example is fecal microbiota transplantation (FMT). Four patients with pseudomembranous enterocolitis were the first to use FMT in modern western medicine, which achieved a good curative effect. As medicine continues to advance, FMT in the treatment of recurrent Clostridium difficile infection has been included in the American clinical guidelines (Surawicz et al., 2013). FMT has now been confirmed to have beneficial effects in many other diseases, such as intestinal graft-versus-host disease (van Lier et al., 2020), irritable bowel syndrome (Johnsen et al., 2018), inflammatory bowel disease, and multi-drug-resistant organisms (Ghani et al., 2021). Liu et al. (2021) administered oral FMT to 11 discharged patients with COVID-19 to analyze the potential effect of FMT on the gut microbiota and immune system after SARS-CoV-2 infection, and all five patients who developed gastrointestinal symptoms were in remission after treatment. FMT modulated gut microbiota disturbances by decreasing the relative abundance of *Proteobacteria* and increasing the relative abundance of *Actinobacteria* at the phylum level, respectively. *Bifidobacterium* and *Faecalibacterium* increased remarkably at the genus level. A case study reported two patients (patient 1: an 80-year-old man with pneumonia/sepsis, patient 2: a 19-year-old man with ulcerative colitis on immunosuppression) with severe risk factors for COVID-19 who underwent FMT due to Clostridioides difficile infection and who were later diagnosed with COVID-19. The most striking observation that emerged from the comparison of the cases was that the clinical symptoms of these two patients were mild. They did not develop into severe COVID-19 and symptoms were even quickly relieved (Bilinski et al., 2022). These researches are only a preliminary clue for FMT to serve as the adjuvant therapy for COVID-19, and further clinical application necessitates additional study. It should be noted that FMT has strict requirements for donor feces, especially during the COVID-19 pandemic. Improper screening and handling of feces may be counterproductive and cause the spread of the SARS-CoV-2 (Kazemian et al., 2021). Experts have formulated clinical medical guidelines for the application of FMT during the COVID-19 epidemic to ensure safety (Ianiro et al., 2020). In general, these results have revealed the potential role for FMT in COVID-19 management.

### Antibiotics

Probiotics, prebiotics, and FMT aim to increase the abundance of beneficial bacteria, while antibiotics are used to suppress the abundance of harmful microbiota species. The WHO proposed that broad-spectrum antibiotics may be required for patients with COVID-19 with possible severe acute respiratory infection (SARI) and sepsis, which can cover as many pathogenic bacteria as possible (World Health Organization, 2020^2^). Randomized data on COVID-19 patients in 38 Michigan hospitals found that at least 50% of patients received early empiric antibiotic therapy (Vaughn et al., 2021). The application of antibiotics in critically ill patients in China also accounts for a large rate (Yang X. et al., 2020; Zhou F. et al., 2020), and plays a key role in inhibiting secondary infection in patients. Conversely, antibiotic therapy can indiscriminately eliminate normal commensal microbiota while eliminating pathogens, leading to dysbiosis of the micro-ecosystem. Antibiotic treatment of COVID-19 patients can have a significant impact on the intestinal microbiota compared to healthy controls, with the availability of fewer commensal bacteria beneficial to host immunity, including *Faecalibacterium prausnitzii, Ruminococcus obeum*, and *Eubacterium rectale* (Zuo et al., 2020b), while *Coprobacillus* and *Clostridium ramosum*, which have been confirmed to be positively correlated with the severity of COVID-19 (Zuo et al., 2020b) increase (Cao et al., 2021). Furthermore, the overuse of antibiotics can lead to increased bacterial resistance (Li J. et al., 2020; Rawson et al., 2020), increasing the burden of disease and death. A cohort study showed that about 50% of antibiotic-treated patients still died from secondary infections of multiple bacteria in the lungs (Du et al., 2020). Especially during the epidemic, the use of antibiotics has faced great challenges. Therefore, the antibacterial treatment of patients with COVID-19 must have clear and strict indications, sensitive antibiotics after pathogenic testing should be carefully selected, and appropriate medication time should be adjusted according to the specific condition of the patient (Sieswerda et al., 2021).

### Auxiliary role of traditional Chinese medicine in COVID-19

Integrative therapy with traditional Chinese medicine (TCM) and Western medicine has achieved reliable results in the treatment of Chen and Nakamura (2004) and H1N1 influenza (Wang et al., 2011). The mechanism of TCM is still unclear as the crystallization of ancient Chinese wisdom. Many studies have used modern medical approaches to explain its complex effects through scientific data and experiments. TCM can regulate the intestinal mucosal barrier, the composition of the gut microbiota and its metabolites, and, vice versa, the gut microbiota can transform TCM components and influence its effect (Feng et al., 2019). SP2-1, a homogeneous polysaccharide isolated from *Scutellaria baicalensis*, significantly increased the abundance of Firmicutes, Bifidobacterium, Lactobacillus, and Rosatello in mice, and inhibited Bacteroidetes and Proteobacteria, compared to the model group, which may be related to relief from colitis (Cui et al., 2021). Glycyrrhizic acid modulated gut microbiota dysbiosis against high-fat diet-induced lung premetastatic niche formation and metastasis (Qiu et al., 2019). The Huanglian Jiedu Decoction ameliorated hyperglycemia by increasing the abundance of SCFA-producing bacteria, reducing the abundance of opportunistic pathogens, and regulating metabolic functions related to the microbiota, such as bile acid synthesis and glycolysis (Chen et al., 2018). The gut microbiota interacts with the components of TCM leading to a series of oxidation, reduction, and further chemical reactions that are responsible for converting the ingredients of TCM such as saponins and polysaccharides into beneficial metabolites, which can improve oral availability (Xu et al., 2017; Zhang F. et al., 2020). Based on these data, we can infer that TCM may enhance immunity, improve pathological conditions, and alleviate complications by interacting with intestinal flora (Chen et al., 2021). The Chinese government has already added TCM treatment to the third edition of guidelines on diagnosis and treatment of COVID-19 (National Health Commission of the People’s Republic of China, 2020^3^), and then Academician Zhang Boli recommended “three drugs and three methods” to treat COVID-19. Currently, TCM prescriptions have been widely applied in China to resist COVID-19 and have definite curative effects (Li L.C. et al., 2020; Yang Y. et al., 2020; Publicity Department of the People’s Republic of China, 2022). The limitation of TCM application is that the active ingredients and their mechanism to alleviate COVID-19 are unclear. Although the mitigation effect is significant, the leverage of TCM is inadequate in countries other than China. The scientific basis needs to be explored to bring TCM to the world stage.

### COVID-19 vaccine

The effectiveness and protection rate of the COVID-19 vaccine has been recognized, and COVID-19 vaccines have been widely available in China to date. Vaccination can improve individual immunity to COVID-19 (Zeng G. et al., 2022). In the long run, convenient and affordable vaccines will also play an important role in overall epidemic control and economic recovery. Substantial studies have confirmed the non-ignorable influence of the microbiota on the immune efficacy of vaccines (Valdez et al., 2014; de Jong et al., 2020; Lynn et al., 2022). Probiotics and prebiotics have been shown to enhance the immunogenicity of influenza vaccines in clinical trials (Lei et al., 2017). A recent study found that specific markers of the gut microbiota were associated with immune responses and adverse events after subjects were vaccinated against COVID-19. For the inactivated vaccine (CoronaVac), the enrichment of *B. adolescentis* and carbohydrate metabolism-related pathways were associated with higher antibody response. For the mRNA vaccine (BNT162b2), the abundance of bacteria with flagella and fimbriae in the baseline gut microbiota was positively associated with higher antibody responses (Ng et al., 2022b). Additionally, animal experiments confirmed the potential of use the oral probiotic strain Lactobacillus plantarum GUANKE in enhancing the specific humoral and cellular immune responses of SARS-CoV-2 vaccines (Xu J. et al., 2021). The findings of these studies have several important implications for future practice and provide a promising direction to improve the immune efficacy of the COVID-19 vaccine.

### ACE2-based therapeutic strategies

**FIGURE 2:**
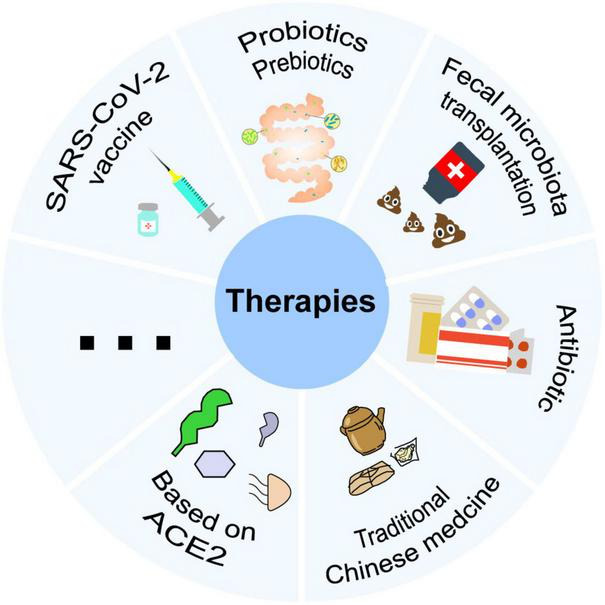
Potential COVID-19 therapies associated with microbiota such as prebiotics and prebiotics, fecal microbiota transplantation, antibiotics, traditional Chinese medicine, SARS-CoV-2 vaccine, and treatment based on ACE2, etc.

ACE2 is the main receptor for SARS-CoV-2 to invade the human body, but it also plays a vital role in maintaining intestinal microecology and intestinal immune regulation. It has been suggested that preventing ACE2 from binding to SARS-CoV-2 may be used to treat COVID-19 (Zhang H. et al., 2020; Penninger et al., 2021). Human recombinant soluble ACE2 (hrsACE2) reduced SARS-CoV-2 recovery in Vero cells by 1,000-5,000-fold and could block the early stages of SARS-CoV-2 infection (Monteil et al., 2020). Palmitoylated ACE2-enriched extracellular vesicles bind to SARS-CoV-2 and protect the host from viral-induced lung inflammation (Xie et al., 2021). A recent study overturned these notions and found that ACE2, an important part of the RAS axis, its impaired catalytic function may be a key factor in the metabolic complications of COVID-19 (Li et al., 2022). ACE2 enzyme activators relieve insulin resistance, reduce inflammatory factors in plasma, improve liver and kidney metabolic functions, and regulate disordered gene functions. Overall, ACE2 enzyme activators ameliorate metabolic defects and inhibit the entry of SARS-CoV-2 after COVID-19 infection. Restoring ACE2 enzymatic activity rather than inhibiting ACE2 levels is critical in circumventing SARS-CoV-2 infection and subsequent metabolic complications. Taken together, ACE2-based therapy is worthy of consideration. Possible treatments for COVID-19 are shown in Figure 2.

## Conclusion

This review provides a deeper insight into the relationship between the microbiota and COVID-19. The respiratory tract, intestinal tract, and oral are susceptible to SARS-CoV-2 infection, and microorganisms at these sites undergo significant changes. Microecosystem disorders widely affect tissues and organs throughout the body through cytokine storms, the destruction of immune barriers, and the inhibition of immune responses. Microbiota metabolites may also modulate immunity through various mechanisms, for example, short-chain fatty acids inhibiting viral infection by reducing ACE2 and TMRSS2 gene expression (Li J. et al., 2021). Furthermore, the microbiota may also interact with ACE2, the receptor for viral entry into the body. The potential of the microbiota as a diagnostic marker for many other diseases has been described. We summarize its potential as a diagnostic marker for COVID-19 and propose a possible non-invasive diagnostic method. Gut dysbiosis persists long after the virus is cleared, with long-term effects on the body. Therefore, the objective of clinical management should be to not only remove the virus but also restore the abnormal intestinal microbiota. In the future, microbiota and microecological therapy are expected to become an important means of intervention in COVID-19 and may play a favorable role in preventing and controlling the global epidemic.

However, due to the rapid variability of SARS-CoV-2 and the uncontrollability of confounding factors, we should also recognize the limitations of current studies. It is difficult to determine whether the changes in the microbiota reported to date are caused by COVID-19 rather than treatment modalities, individual differences, diet, etc. Meanwhile, the mechanisms underlying the impact of the microbiota on the severity and susceptibility of COVID-19 remain to be clarified. Considerably more work is required to determine the mechanisms by which the microbiome modulates host immunity and affects acute COVID-19 and long COVID, thereby opening perspectives for new ideas for the treatment and prevention of COVID-19.

COVID-19 vaccines such as inactivated vaccines, live attenuated vaccines, recombinant protein vaccines, and mRNA vaccines have been gradually rolled out in the global population and have shown good performance in preventing SARS-CoV-2 infection, which can effectively reduce the risk of infection, severe illness, and death (Jara et al., 2021; Rearte et al., 2022). However, the efficacy and duration of protection after vaccination varies in different populations (Goel et al., 2021). Studies showed specific gut microbiota markers associated with improved immune responses and reduced adverse events following the COVID-19 vaccine (Ng et al., 2022a). Hence, studying the interaction of microbiota with host immunity can help us develop microbiota interventions to improve vaccination outcomes.

To date, more than 500 million people have been infected with COVID-19, and some of these patients have recovered with long-term complications of COVID-19, such as extreme fatigue, decreased memory and concentration, and decreased sense of taste and smell, the exact cause of which remains unclear. Liu Q. et al. (2022) found gut microbial alterations in patients with post-acute COVID-19 syndrome, and gut microbiota at admission is associated with the occurrence of post-acute COVID-19 syndrome. Therefore, we should further explore the regulation of the gut microbiota to promote recovery in patients with long-term COVID-19 syndrome in the future.

Existing studies have focused mainly on the effects of wild-type SARS-CoV-2 infection on host microorganisms. However, the Omicron variant has now replaced the wild-type and Delta variant and is widely disseminated around the world (Karim and Karim, 2021). Furthermore, Omicron variants can evade infection and vaccination-induced antibodies, which undoubtedly exacerbates the risk of infection (Callaway, 2021; Cele et al., 2022). The effect of Omicron on the host microbiota and the difference between the effects of Omicron and wild-type SARS-CoV-2 on host microbiota are still unclear. Therefore, future research should probably also focus on addressing these concerns.

## Author contributions

SZ and XX proposed the study. JG and HW performed the research and wrote the first draft. All authors contributed to interpretation of the study and to further drafts.

## Footnotes

1
https://www.fda.gov/medical-devices/coronavirus-covid-19-and-medical-devices/sars-cov-2-viral-mutations-impact-covid-19-tests

2
https://apps.who.int/iris/handle/10665/331446?locale-attribute

3
http://www.nhc.gov.cn/xcs/zhengcwj/202001/f492c9153ea9437bb587ce2ffcbee1fa.shtml
